# Saccadic Adaptation Alters the Attentional Field

**DOI:** 10.3389/fnhum.2016.00568

**Published:** 2016-11-16

**Authors:** Farahnaz A. Wick, Tyler W. Garaas, Marc Pomplun

**Affiliations:** Visual Attention Laboratory, Department of Computer Science, University of Massachusetts Boston, BostonMA, USA

**Keywords:** attentional field, saccadic adaptation, eye movements, spatial attention, flanker task

## Abstract

It is currently unknown whether changes to the oculomotor system can induce changes to the distribution of spatial attention around a fixated target. Previous studies have used perceptual performance tasks to show that adaptation of saccadic eye movements affects dynamic properties of visual attention, in particular, attentional shifts to a cued location. In this study, we examined the effects of saccadic adaptation on the static distribution of visual attention around fixation (attentional field). We used the classic double step adaptation procedure and a flanker task to test for differences in the attentional field after forward and backward adaptation. Reaction time (RT) measures revealed that the shape of the attentional field changed significantly after backward adaptation as shown through altered interference from distracters at different eccentricities but not after forward adaptation. This finding reveals that modification of saccadic amplitudes can affect metrics of not only dynamic properties of attention but also its static properties. A major implication is that the neural mechanisms underlying fundamental selection mechanisms and the oculomotor system can reweight each other.

## Introduction

Our visual system is limited in its capacity to process information. Nevertheless, we can quickly locate our favorite shirt in a cluttered closet, react and catch a ball thrown at us and read a signboard across a busy street—all these tasks require breaking down crowded visual input into manageable parts. The visual system compensates for its limited information processing capacity using selective visual attention, the main mechanism by which our visual input is filtered so we can consciously perceive information relevant to current behavior. We attend to points of interest in our visual field and thereby prioritize visual processing near that location; moreover, we typically switch attended locations several times per second by covertly or overtly *shifting* attention to the next point of interest. Abundant psychophysical evidence shows that covert and overt shifts of attention (i.e., saccadic eye movements) are closely linked; attention shifts to the landing position of a saccade prior to the saccade onset (Kowler et al., 1995; Deubel and Schneider, 1996; Moore and Fallah, 2001; Schneider and Deubel, 2002). The targeting of attentional shifts can be classified as a dynamic property of attention, whereas the distribution of attention while fixating on a point or object can be considered its static property. While there is a large body of research addressing dynamic (Krose and Julesz, 1989; Hoffman and Subramaniam, 1995; Deubel and Schneider, 1996; Itti and Koch, 2000; Carrasco and McElree, 2001; Nothdurft, 2002; Schneider and Deubel, 2002) and static properties (Eriksen and Yeh, 1985; Eriksen and James, 1986; LaBerge and Brown, 1986; Lavie, 1995; Intriligator and Cavanagh, 2001; Müller et al., 2005) of attention separately, little is known about the relationship between these two characteristics.

In the experiments reported here, we exploited the plasticity of oculomotor programming to test whether mechanisms controlling saccadic eye movements affect the attentional distribution around a fixation point (or the *static attentional map*, also known as the attentional field). Specifically we used a process that modifies the amplitude of saccadic eye movements called saccadic adaptation. Saccadic adaptation is a form of motor learning that enables the oculomotor system to maintain accurate saccade targeting under varying conditions induced by fatigue, aging, or injury. Neural mechanisms evaluate the visual error between the actual post-saccadic landing position and its intended target and adjust the saccadic vector to reduce this error in future saccades (McLaughlin, 1967; Wallman and Fuchs, 1998; Noto and Robinson, 2001).

Previous research using the paradigm of saccadic adaptation has revealed behavioral evidence for the spatiotemporal correspondence between overt eye movements and the dynamic attentional map. Several studies report that during saccadic adaptation the pre-saccadic attentional shift adapts correspondingly (McFadden et al., 2002; Doré-Mazars and Collins, 2005; but see Ditterich et al., 2000a,b). In these studies, when saccadic amplitude along a vector was reduced or increased using saccadic adaptation, the pre-saccadic focus of attention followed the saccadic vector directly. A recent study by Khan et al. (2014) has shown that the locus of attention itself can act as an error signal for saccadic adaptation even in the presence of a clear target location.

The above work provides evidence that dynamic attentional processes, involved with saccades and their landing positions, share spatial maps with saccade control centers (see Corbetta and Shulman, 2002). Recent studies have shown that saccadic adaptation can induce perceptual changes (Zimmermann and Lappe, 2010; Garaas and Pomplun, 2011) and that the visual component of adaptation modifies spatial maps in response to saccade error signals (Zimmermann et al., 2011). When these spatial maps are distorted through saccadic adaptation (Collins and Doré-Mazars, 2006; Collins et al., 2007; Zimmermann and Lappe, 2009), the dynamic attentional map appears to get distorted as well. If dynamic properties of attention are affected by distortions in these spatial maps, then it is possible that static properties of attention could be affected similarly by saccadic adaptation, as the same spatially selective mechanism is at work (Kowler et al., 1995; Moore and Fallah, 2004). To test this hypothesis, in the present study, we adapted observers’ saccadic eye movements and investigated whether the oculomotor map and the static attentional map were distorted in corresponding ways.

We employed a variant of the flanker paradigm used by Müller et al. (2005) to measure the spread of the static attentional map before and after adaptation. Eriksen and his colleagues (Eriksen and Eriksen, 1974; Eriksen and Yeh, 1985; Eriksen and James, 1986) proposed that the distribution of attentional resources around a focal point and the spatial extent of this region have a reciprocal relationship like that of the power of a zoom lens camera, that is, attentional resources decrease with retinal eccentricity. They tested this distribution using a flanker task (Eriksen and Eriksen, 1974)—a common paradigm used to estimate the spatial extent to which processing of irrelevant information takes place. The classic flanker effect is shown through differences in reaction times to a target stimulus when distracters are presented spatially close to it due to their interference with target processing and response initiation. The advantage of the flanker paradigm over others is that attention can be anchored at a particular point and the interference from distracters within the static attentional map can be measured indirectly through degradation of reaction times. However, the disadvantage of this paradigm is that it produces noisy data and requires a large number of trials to capture the effect, making it difficult to detail minute spatial changes in the attentional field. Therefore, the aim of the present study was not to determine the exact shape or size of attentional fields, but to understand the relationship between attentional fields and oculomotor programming.

In the flanker task of this study, participants knew in advance the position and size of the target whose identity they would have to report. Therefore, their attention was assumed to be anchored on a small display area around their fixation point and their attentional resources would drop with greater retinal eccentricity (Eriksen and Eriksen, 1974; Eriksen and James, 1986; Lavie, 1995; Müller et al., 2003, 2005; Caparos and Linnell, 2010). There were distracters presented at three different eccentricities from the target. We expected distortions in this static map of attention as a result of corresponding distortions in the oculomotor and spatial maps created through saccadic adaptation. Specifically, we hypothesized that the map would contract when the amplitude of saccades decreased (via backward adaptation).

As noted above, the chosen experimental paradigm did not allow us to precisely measure the pre- or post-adaptation shape of the attentional map, i.e., the distribution of attentional resources, but only estimate it at a few points. Therefore, there are two possible scenarios, depending on the initial distribution and the extent of its contraction: (1) if a larger amount of resources lands at the nearest distracter after contraction: interference from the nearest distracter should then be greater than in the baseline condition, while the interference from the other distracters would decrease, (2) if the baseline map is focused tightly by the target position, it could contract away from the nearest distracter: interference from distracters at all eccentricities would then decrease, as indicated by shorter reaction times when compared to the baseline.

Conversely, we anticipated the static map to widen when saccadic amplitude increased (via forward adaptation). Reasoning as above, there are two corresponding possibilities: (1) interference from the nearest distracter could decrease from the baseline while the interference increases at the other eccentricities as the pool of attentional resources is shifted toward larger eccentricities, (2) interference from all distracters could increase, which would result in stronger interference, i.e., longer reaction times, at all distracter eccentricities.

## Materials and Methods

### Participants

Fifty-four right handed healthy adult volunteers (age range: 18–25 years) with reported normal and corrected-to-normal visual acuity and color vision were either paid $30 each or given research credits for their participation in a two-session experiment. All participants were naïve to the hypothesis and the authors did not participate in the experiments. Experimental procedures were approved by the University of Massachusetts Boston Institutional Review Board. Prior to data collection, all participants signed an informed consent form.

### Stimuli and Procedure

**FIGURE 1:**
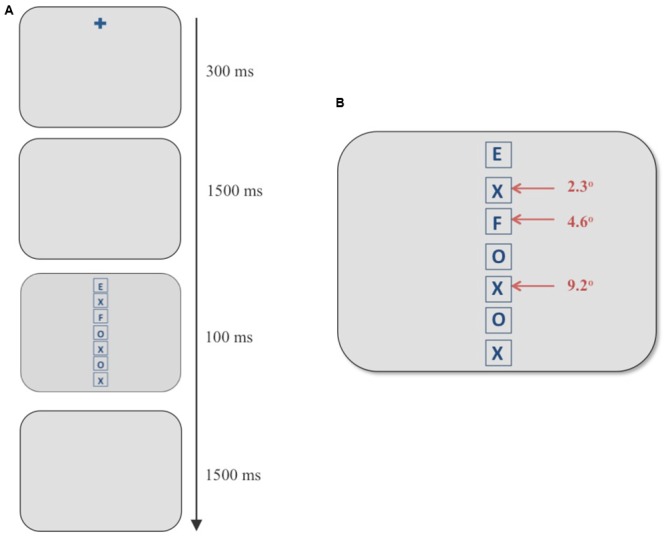
**(A)** Example of a discrimination trial using the flanker paradigm. **(B)** Stimulus screen in the discrimination task consisting of a vertical column of seven boxes. The target always appeared in the topmost box and an incompatible distracter appeared at any of the three eccentricities marked with red arrows. In the example above, an “F” (an incompatible distracter) appears at an eccentricity of 4.6° when the target is an “E.”

**FIGURE 2:**
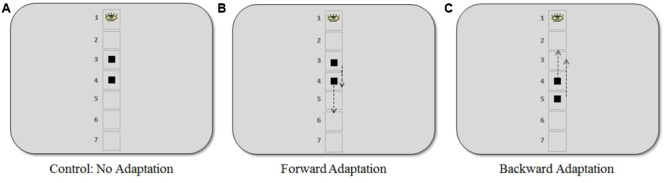
**Positions where the target appeared in the saccadic adaptation procedure for the three experiments. (A)** In the control experiment, the target appeared at visual angles of 4.6° and 6.9° (positions 3 and 4, respectively). **(B)** In the forward adaptation experiment, the target appeared at 4.6° (short saccade) or 6.9° (long saccade). **(C)** In the backward adaptation experiment, the target appeared at 6.9° (short saccade) or 9.2° (long saccade). The arrows indicate the approximate locations (50% step) where the target moved when a saccade was in progress. Note that the placeholder squares or numbering were not present during the adaptation task.

Stimuli were presented on a 24-inch ViewSonic V3D245 LCD monitor using a resolution of 1024 × 768 pixels and a refresh rate of 100 Hz. Participants sat approximately 65 cm from the screen resulting in a horizontal and vertical viewing angle of 34° and 25°, respectively. Eye movements from the right eye were recorded using the SR Research Ltd, EyeLink 1000 eye tracker system. The average error of visual angle in this system was roughly 0.5° and the sampling frequency was 1000 Hz. The stimulus configuration and experimental procedures used are shown in **Figures 1** and **2**.

Participants were scheduled to participate in two separate sessions on two consecutive days, approximately around the same time each day. They were seated in a dimly lit room with their head stabilized on a chin and forehead rest, with instructions not to move their head from the chin rest during the experiment. Eye movements were recorded for both sessions.

The first session (“*Session 1*”) lasted approximately 35 min and consisted of a discrimination task. There were six blocks, each block consisting of 100 trials followed by a break during which the participants were instructed to keep their eyes closed. Therefore, each eccentricity was sampled 200 times. The second session (“*Session 2*”), which lasted approximately 50 min, consisted of 100 adaptation trials followed by six blocks of the discrimination task (100 trials per block) interleaved with 30 adaptation trials in between blocks (250 adaptation trials total). After every block of the discrimination task, participants had the option to take a short break during which they were instructed to keep their eyes closed without moving their heads from the chin rest.

#### Discrimination Task

In this task, participants viewed a vertical, centered column of seven letters composed of Es, Fs, Xs, and Os in placeholders (square frames subtending 2° in height and width; see **Figure 1**). The experimental design was adapted from Müller et al. (2005). The letters were blue (average luminance 11 cd/m^2^) against a gray (average luminance 83 cd/m^2^) background. Participants were asked to report the letter in the topmost box in the column (“E” or “F”) using labeled keys of a keyboard. The stimulus screen with letters was presented for 100 ms and was subsequently replaced by a blank gray screen. The participants had 1500 ms to respond before the next trial began automatically. They were instructed to respond as quickly and accurately as possible with no feedback provided. Taking the top box at 0°, each box containing the letters was placed at 2.3° increments with neighboring boxes being 0.3° apart. In each trial, a single incompatible distracter (an “E” if the target was an “F” and vice versa) was presented at one of the three eccentricities marked with red arrows in **Figure 1**, whereas all other locations contained neutral letters (Xs and Os). The independent variable of interest was the eccentricity of the incompatible distracter from the target. Three eccentricities were chosen for the incompatible distracter: the position next to the target (visual angle 2.3°), the third position (visual angle 4.6°), and the fifth position (visual angle 9.2°).

We used a centered vertical design to prevent hemispheric asymmetries in saccadic adaptation from acting as a confounding variable. Recent fMRI studies (Gerardin et al., 2012; Panouillères et al., 2014) have shown that direction of saccadic adaptation in the horizontal plane activates the contralateral and ipsilateral hemisphere in different manners.

#### Adaptation Task

Participants were randomly assigned to one of three different experiments: (1) *control: no adaptation* (16 participants), (2) *forward adaptation* (15 participants), and (3) *backward adaptation* (18 participants). During the adaptation phase of the experiment, a gaze-contingent stimulus manipulation was used to induce post-saccadic visual error. Participants were asked to fixate on a black fixation cross (average luminance 5 cd/m^2^) against a gray background (average luminance 83 cd/m^2^) that was positioned approximately in the same location as the center of the top box in the discrimination task. Note that there were no boxes or outlines from the discrimination task present during adaptation trials. After a random delay between 500 and 1000 ms, the target, a black square (visual angle 0.7° × 0.7°) appeared and participants were asked to make an eye movement immediately to the square. During their saccade, the square either moved forward, backward or not at all according to the adaptation procedure for each experiment described below (see **Figure 2**).

The control experiment was conducted to determine a baseline measure. The square target appeared with equal frequency at visual angles 4.6° or 6.9° and did not move during the saccade. In the forward adaptation experiment, the square appeared at either 4.6° (“*short saccade*”) or 6.9° (“*long saccade*”) below where the target appeared in the discrimination task. Finally, in the backward adaptation experiment, the square appeared at 6.9° (“*short saccade*”) or 9.2° (“*long saccade*”).

The square target, in the adaptation task, would move 50% forward or backward of the initial target eccentricity when a saccade was made toward it (see **Figure 2**). The step size used in our experiments was larger than what is typically used in the literature. However, previous work in our lab (Garaas et al., 2008; Garaas and Pomplun, 2011) and by others using a 40% step size (Albano, 1996; Zimmermann et al., 2011) indicates that such large steps can be an effective way to induce substantial changes in saccade amplitude that are necessary for the purpose of the present study. As Pélisson et al. (2010) note, changes observed during adaptation are independent of any conscious detection of the target step, meaning that adaptation can still be induced even if participants noticed the step, which occasionally happened in the present study. Saccades were detected online and display change was triggered when the gaze passed outside a virtual circle of 2° radius centered on the fixation point. In the oﬄine analysis, only eye movements whose velocity exceeded 22°/s and whose acceleration exceeded 4000°/s^2^ were included (criteria based on Schnier and Lappe, 2011). Furthermore, only saccades whose direction did not deviate by more than 14° from the direction of the post-saccadic target position were considered. The maximum delay between the detection of a saccadic eye movement and the display change was 12 ms.

An equal number of short and long saccade trials (125 trials of each kind) appeared during the adaptation blocks across experiments. We used different target eccentricities to prevent predictability of target location and any strategies participants might use to complete the task. Different pre-saccadic target positions for forward and backward adaptation were used to keep saccade amplitudes within similar ranges during the adaptation processes.

Participants were given written and verbal instructions before each session in which they were only informed about the task they had to perform, and they were not aware of the experimental manipulations at the distracter locations for each experiment. They completed 15 practice trials of the discrimination task before starting Session 1 and 15 practice trials of the adaptation task prior to Session 2. In order to ensure fixation and covert alignment of attention during the discrimination task, eye movements were recorded and any trials where the gaze position was outside a 2° radius of the fixation point during the 1500 ms gap period or during stimulus presentation were discarded.

## Results

**FIGURE 3:**
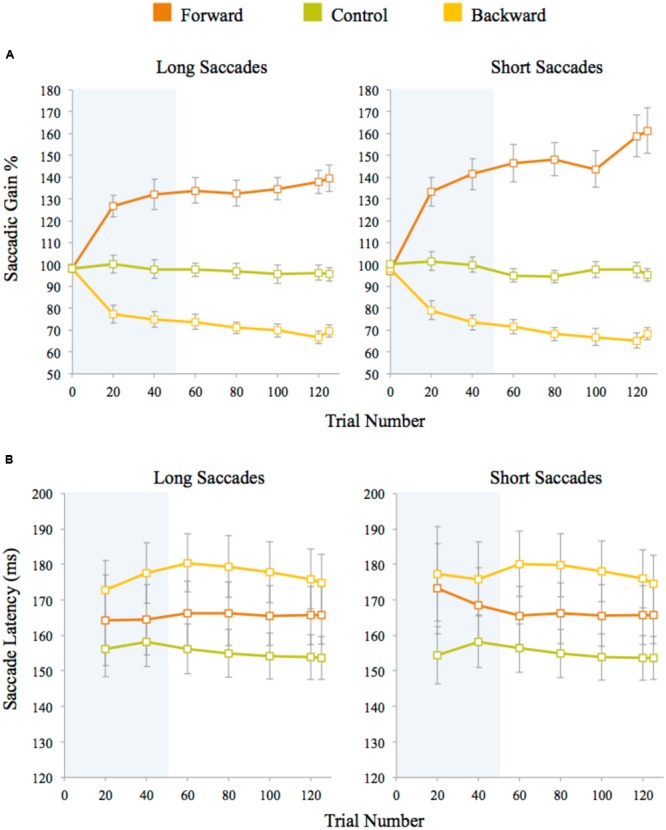
**(A)** Saccadic amplitude gain for long and short saccades averaged across trials for each experiment. **(B)** Saccade latencies measured after target presentation in adaptation trials. Latencies for long and short saccades were averaged across trials for each experiment. Twenty trials were averaged for each data point (except for the last data point which is an average of five data points only). The error bars represent standard error. The shaded area represents the adaptation block before the discrimination blocks began.

Saccadic gain during the adaptation task was measured for the primary saccades that started within 2° of visual angle of the initial fixation marker and whose direction did not deviate by more than 14° from the direction of the post-saccadic target. Approximately 2% adaptation trials were discarded on average for each participant using this criterion. The percentage of gain was calculated by (saccade amplitude/pre-saccadic target eccentricity) × 100. A plot of saccadic gain averaged across trials for the three experiments is given in **Figure 3A**. In the control experiment, the average gain was approximately 97% for both long and short saccades. The gain measured after the initial 100 adaptation trials for forward adaptation was approximately 132% for long saccades and 141% for short saccades. For backward adaptation, this value was approximately 75% for both long and short saccades. We analyzed saccade latencies to check whether participants used any cognitive strategies during adaptation procedure and found similar saccade latencies within each experiment, indicating that no strategies were employed during the adaptation task (see **Figure 3B**). The saccadic latency means differ across experiments by approximately 10 ms for both long and short saccades. These differences were not significant as shown by a one-way ANOVA of the average latency between the experiments for both long saccades, *F*(2,48) = 2.57, *p* = 0.09, and short saccades, *F*(2,48) = 2.53, *p* = 0.09. Saccadic latencies across the experiments were within the normal reported range (120–200 ms) for fast regular saccades.

In the discrimination task, we analyzed trials in which participants correctly identified the target letter. Data from a participant was used only if their accuracy was within two standard deviations of the mean performance and they experienced sufficient saccadic adaptation in Session 2, i.e., the saccadic amplitudes were at least 10% greater or smaller for forward or backward adaptation, respectively, as compared to their pre-adaptation amplitude. We set these thresholds to ensure that any changes observed in the attentional map were not due to tradeoffs in accuracy. Data from five participants were discarded; four participants showed below-threshold performance in the discrimination task and one participant did not experience sufficient forward adaptation. The independent variables were Eccentricity (three visual angles: 2.3°, 4.6°, 9.2°) and Session (1: before and 2: after the adaptation task). The dependent variable was average reaction time for each participant. It is typical to use reaction times instead of accuracy in studies involving the flanker task (Eriksen and Eriksen, 1974; Eriksen and James, 1986; Müller et al., 2003, 2005). To minimize influence from outliers, any trials in which the reaction time deviated from the mean by more than two standard deviations were discarded for each participant. On average 1% of the discrimination trials with correct responses were discarded for each participant using this criterion.

**FIGURE 4:**
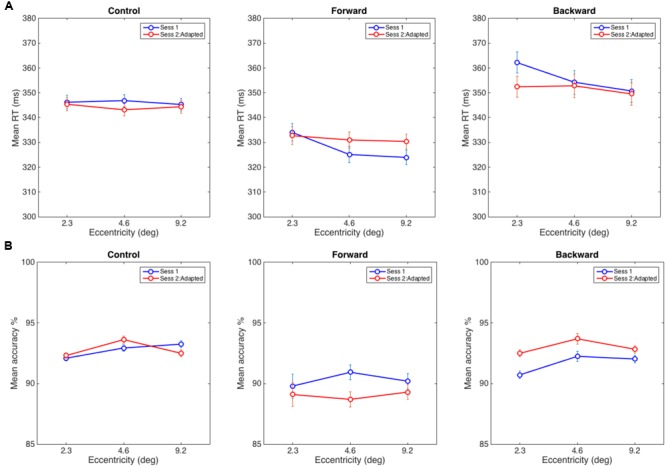
**(A)** Average reaction time in the discrimination task in Control (left), Forward (middle), and Backward (right) adaptation experiments. **(B)** Mean accuracy at reporting a target when distracters appeared at eccentricities of 2.3, 4.6, and 9.2°. The error bars represent within-subject error computed using the procedure in Cousineau (2005).

A 3 (Eccentricity) × 2 (Session) repeated measures ANOVA was used to analyze average RTs (see **Figure 4A**). According to our hypothesis, we did not expect to find a significant interaction in the control experiment because the static attentional map should not be distorted without any adaptation taking place. The results were in line with this expectation—we did not find any significant main effect or interaction in the control experiment—interaction of Eccentricity and Session: *F*(2,30) = 0.95, *p* = 0.40; Eccentricity: *F*(2,30) = 0.281, *p* = 0.76; Session: *F*(1,15) = 0.134, *p* = 0.72. Note that the RT slopes are flat in both sessions, and we did not find the eccentricity effect in the flanker task as reported in literature, Session 1: *F*(2,30) = 0.4, *p* = 0.67 and Session 2: *F*(2,30) = 0.73, *p* = 0.50. This could be a characteristic of the group of the participants in the control experiment as RT data is noisy and the flanker effect requires a large number of trials (for example: 1024 times per eccentricity in Müller et al.’s (2005) experiments). Although note that in Eriksen and Eriksen’s (1974) seminal work, they demonstrated the eccentricity effect using 24 samples per eccentricity but over a smaller and different range of eccentricities. The critical measure in our experiments is the interaction between the two sessions which was not significantly different in the control condition, as expected.

As hypothesized, in the forward adaptation experiment, there were two possible interference patterns by distracters that could indicate a widening of the static attentional map after adaptation: (1) interference from the nearest distracter (2.3°) decreases while the interference at the other two eccentricities increase if attentional resources are shifted toward farther eccentricities, or (2) interference from distracters at all eccentricities increase. The data suggests neither (1) or (2): the interference at the nearest eccentricity did not decrease from the baseline but the interference from the other two eccentricities increased. The eccentricity effect for the flanker task was present in Session 1, *F*(2,28) = 7.39, *p* < 0.005 but not in Session 2, *F*(2,28) = 0.79, *p* = 0.47. The interaction of Eccentricity and Session was close to the significance threshold, *F*(2,28) = 3.03, *p* = 0.06, and a main effect of Eccentricity, *F*(2,28) = 7.83, *p* < 0.005; however, there was no main effect for Session, *F*(1,14) = 0.283, *p* = 0.60. A 2 (Eccentricity) × 2 (Session) RM-ANOVA between the distracter at 2.3° with distracters at other positions showed that interaction with the farthest distracter (visual angle 9.2°) was significant, *F*(1,14) = 6.08, *p* < 0.05 and the interaction with the distracter at 4.6° was not, *F*(1,14) = 3.00, *p* = 0.11 (see **Figure 4A**).

In the backward adaptation experiment, we expected the attentional map to contract after adaptation. Since we could not estimate how far the map would contract, there were two possibilities: (1) the nearest distracter (2.3°) causes more interference than the other two distracters if it receives more attentional resources after adaptation or (2) the attentional map contracts beyond the nearest distracter and thus interference would decrease for all distracter eccentricities. We expected this pattern since near-target distracters received the most pre-adaptation attentional resources and should show reduction in post-adaptation interference. The baseline eccentricity effect for the flanker task was present in Session 1, *F*(2,34) = 20.86, *p* < 0.0005 but not in Session 2, *F*(2,34) = 2.96, *p* = 0.07. There was a significant interaction of Eccentricity and Session, *F*(2,34) = 7.73, *p* < 0.005 and a main effect of Eccentricity, *F*(2,34) = 21.3, *p* < 0.001 and no significant effect for Session, *F*(1,17) = 0.21, *p* = 0.65. Again, the data did not show support for hypothesis (1) or (2). A 2 (Eccentricity) × 2 (Session) RM-ANOVA showed a significant interaction between distracters at 2.3° and 4.6°, *F*(1,17) = 9.9, *p* < 0.007 and between distracters at 2.3° and 9.2°, *F*(1,17) = 11.2, *p* < 0.005. The observed effects were due to a reduction of interference between sessions that was more pronounced for the nearest distracter than for the other two distracters (see **Figure 4A**).

Note that the hypotheses for the adaptation conditions were calculated speculations based on previous literature (Müller et al., 2005). Unfortunately, the current experiments do not allow us to exactly measure the spread of the attentional field before and after adaptation, which in turn makes it impossible to predict *precisely* how the attentional field is modulated. However, these results are plausible if one considers the attentional spread in the current task to have a narrow Gaussian profile (Reynolds and Heeger, 2009).

In order to show that the attentional field changed significantly between the adaptation and the control experiments, we did a 3 (Group) × 3 (Eccentricity) × 2 (Session) RM-ANOVA. There was a significant interaction between Group, Eccentricity and Session, *F*(4,92) = 2.7, *p* < 0.05. This interaction shows that interference patterns observed in the adaptation and control experiments are significantly different between the sessions. To understand whether the direction of adaptation affected the attentional field, we looked at the interaction between the control and forward adaptation experiments. A 2 (Group) × 3 (Eccentricity) × 2 (Session) RM-ANOVA revealed a three-way significant interaction between Group, Eccentricity and Session, *F*(2,58) = 3.20, *p* < 0.05, showing that the interference pattern observed is different from the control group. Similarly, a 2 (Group) × 3 (Eccentricity) × 2 (Session) RM-ANOVA between the control and backward adaptation experiments revealed a significant three-way interaction, *F*(2,64) = 5.78, *p* < 0.005. A 2 (Group) × 3 (Eccentricity) × 2 (Session) RM-ANOVA between the forward and backward adaptation experiments showed no significant three-way interaction, *F*(2,62) = 0.03, *p* = 0.97. These results indicate that the adaptation affects the attentional field under the constraints of the given task but it is unclear whether the direction of adaptation (forward vs backward) causes significantly different patterns of interference.

In order to ensure that the RT data were not biased by a speed-accuracy tradeoff, we also analyzed the accuracy of responses. For the control experiment, we did not find any significant interaction or main effects: interaction of Eccentricity and Session: *F*(2,30) = 2.22, *p* = 0.16; Eccentricity: *F*(2,30) = 1.89, *p* = 0.19 and Session: *F*(1,15) = 0.02, *p* = 0.90. Similarly, the forward adaptation experiment did not reveal any such effects: interaction of Eccentricity and Session: *F*(2,28) = 1.14, *p* = 0.33, Eccentricity: *F*(2,28) = 0.19, *p* = 0.83 and Session: *F*(1,14) = 0.836, *p* = 0.38. However, for backward adaptation, there were main effects for Eccentricity: *F*(2,34) = 3.49, *p* < 0.05 and Session: *F*(1,17) = 6.27, *p* < 0.05 but there was no significant interaction, *F*(2,34) = 0.78, *p* = 0.47 (see **Figure 4B**).

Given this significant difference in accuracy in the backward adaptation experiment, we adjusted the average RTs to account for this tradeoff by dividing the RTs by accuracy. The results did not change with the adjusted RTs, a significant interaction of Eccentricity and Session: *F*(2,34) = 4.94, *p* < 0.02; a main effect of Eccentricity: *F*(2,34) = 13.4, *p* < 0.001 and no significant effect for Session: *F*(1,17) = 1.19, *p* = 0.29. The accuracy trends observed in the backward adaptation experiments were most likely due to practice effects.

Similarly, we report the results for adjusted RTs for the forward adaptation experiment. There was no significant interaction of Eccentricity and Session: *F*(2,28) = 2.14, *p* = 0.13; a main effect for Eccentricity: *F*(2,28) = 3.61, *p* < 0.05 and no significant effect for Session: *F*(1,14) = 2.23, *p* = 0.16. For the control experiment, there was no significant interaction of Eccentricity and Session: *F*(2,30) = 2.74, *p* = 0.14; no significant effect for Eccentricity: *F*(2,30) = 2.64, *p* = 0.09 or Session: *F*(1,15) = 0.16, *p* = 0.70.

In summary, the overall pattern of results indicates that saccadic adaptation seems to affect the static attentional map and this effect is observable noticeably in the backward adaptation experiment.

## Discussion

The error signals utilized by saccadic adaptation must incorporate prediction and selection mechanisms. Attention is intimately linked to prediction, selection, and saccades (Posner, 1980; Desimone and Duncan, 1995; Kowler et al., 1995; Deubel and Schneider, 1996; Castet et al., 2006) and there is evidence that attention itself can act as the error signal for adaptation in a situation where the target remains in its pre-saccadic position during adaptation and salient distracters are presented to capture attention (Khan et al., 2014). The topography of the spatial distribution of attention is a critical component to many theoretical models of visual attention including the spotlight (Posner, 1980), zoom lens (Eriksen and Yeh, 1985), and normalization (Reynolds and Heeger, 2009) models. Prior to these experiments, it was unknown whether static spatial attentional mechanisms, such as the attentional field, are affected by saccadic adaptation and our results provide an initial insight into the nature of this relationship. As discussed in the Section “Introduction,” previous work on the interaction of adaptation and attention provides evidence that adaptation affects dynamic attentional processes. In contrast to previous studies, which investigated the effects of adaptation on only the dynamic attentional map, we studied the effects of saccadic adaptation on the static attentional map.

We found that the static attentional map appears to be distorted after saccadic adaptation. Specifically, using the flanker paradigm, the results showed that the reduction of saccade amplitude led to decreased interference from nearby distracters, suggesting that the static attentional map contracted after saccadic amplitude was reduced. Additionally, after increasing saccadic amplitude through forward adaptation, we observed that interference from distracters grew, with an emphasis on the greatest eccentricity, though this effect is much weaker than in the backward adaptation experiment. While the results did not provide clear evidence for either of our specific hypotheses, they are compatible with our basic assumption that the attentional field expands and shrinks with forward and backward adaptation, respectively. Let us assume that prior to adaptation the distribution of attention follows a tight two-dimensional Gaussian function around the target position. Consequently, there is some interference from the closest distracter but only very small interference from the other two distracters. After forward adaptation, if the attentional field spreads out, we would expect its Gaussian distribution to become wider and flatter, which may cause little change in interference at the closest distracter but increased interference at the farther ones. Similarly, if backward adaptation makes the distribution narrower and taller, it may reduce interference even for the closest distracter. The two other distracters, who did not exert substantial interference before adaptation, would remain at roughly the same level of interference. This explanation is clearly speculative but plausible and provides a starting point for further studies of this issue. An alternate explanation is that adaptation could have perceptual effects as shown by Garaas and Pomplun (2011), and after backward or forward adaptation, participants could perceive the distracters as being closer to or farther from the target, respectively, than they really are. This alternate explanation would still lead to the same line of reasoning used in our experiments. At the very least, our findings imply that neural mechanisms involved in both static and dynamic attention may share spatial maps, as distortions in these maps through saccadic adaptation seem to affect both static and dynamic properties of attention in a similar manner.

As seen in the Section “Results” and **Figure 4A**, the interaction between the session and eccentricities for forward adaptation experiment was close to the threshold for significance (*p* = 0.06). When the RT data was adjusted for performance, the interaction between the session and eccentricities was not significant. Evidence from literature suggests that backward and forward adaptations rely on different mechanisms (Ethier et al., 2008; Panouillères et al., 2012). Forward adaptation or adaptive saccade lengthening has been shown to have a slower time course with smaller gain modifications than backward adaptation (Zimmermann and Lappe, 2010). In the present study, we did not observe the slower time course for adaptation for forward adaptation when compared to backward adaptation (seen in **Figure 3A**). The strong forward adaptation observed could be a consequence of the large step size (50%) used and individual differences of the participants across experiments. It is unclear at this point if the lack of a significant difference in the forward adaptation experiment is due to the noise in the RT signal associated with the flanker paradigm or noise in the adaptation process itself.

Note that the present results do not indicate that total interference from distracters increased or decreased due to adaptation in these experiments as that would be observed through a significant effect of Session. Instead, our data show that the pattern of interference at different eccentricities changed after backward adaptation. In the discrimination task, we used a flanker paradigm to anchor attention to a target spatially flanked by task-relevant distracters. The stimulus was presented for 100 ms, too short to elicit shifts in attention. This level of selection is perceptual and spatial in nature and possibly involves a pool of attentional resources focused on the target location (LaBerge, 1983; Eriksen and James, 1986; Yantis and Johnston, 1990) and we can assume that this pool of resources remained constant in our experiments. We found, along with numerous previous studies (Eriksen and Eriksen, 1974; Eriksen and Yeh, 1985; Eriksen and James, 1986; Müller and Kleinschmidt, 2004), that the strongest interference was usually from the distracter closest to the target. These studies have also shown that the interference from distracters generally decreases with target eccentricity. Since data from the flanker paradigm is noisy and requires many trials, the shape of the pre-adaptation curves and even the RT range vary slightly among the three experiments (especially the control experiment) of the current study. Previous research studied alterations in the shape of the static attentional map by manipulating perceptual and cognitive load and found that the attentional resources can focus or defocus depending on the given task (Macdonald and Lavie, 2008; Caparos and Linnell, 2010). Note that the factors that are known to affect static attentional maps, such as stimulus density, locations, and task difficulty, were kept constant in Sessions 1 and 2 of the discrimination task. This means that the shape alterations observed in our experiments were likely due to saccade amplitude modifications through adaptation.

To understand functional significance of the interaction between saccadic adaptation and attentional field, let us consider the underlying neural basis. Functional imaging studies have defined a frontoparietal cortical network that is active during spatial attention tasks (Heinze et al., 1994; Corbetta, 1998; Szczepanski et al., 2010). It has also been shown that all three major sites of activation for attention (intraparietal, postcentral, and precentral) are simultaneously active during eye movements. These sites of activation have been found to contain topographic representations of visual space involved in controlling attentional operations throughout the visual field, and the same neurons that show pure attentional modulations can also code oculomotor parameters in these areas (see Silver and Kastner, 2009 for a review). Previous studies have shown that saccadic adaptation not only distorts spatiotopic maps for eye movement control (Zimmermann et al., 2011) but also affect perceptual properties such as object perception (Garaas and Pomplun, 2011) or localization (Moidell and Bedell, 1988; Bahcall and Kowler, 1999a,b; Awater et al., 2005; Zimmermann and Lappe, 2009). These distortions in visual perceptual space and perceptual properties might be reflected in these higher-order topographic maps and additionally affect attentional modulation during fixations.

Our data indicates that distortions in these topographic maps clearly bias attentional modulation. However, the current experimental setup and lack of a significant interaction between the forward and backward adaptation experiments does not yield statistically significant support that the pattern of modulation has definitive directional component. Future studies could repeat these experiments using a within-subject design, i.e., mapping changes in the static attentional map of the same subject after they underwent forward and backward adaptation in separate sessions. Moreover, a larger number of experimental sessions could be administered to measure the shape of the attentional maps more closely and allow the testing of direction of modulation.

## Conclusion

In summary, this study showed the involvement of oculomotor control in the spatial deployment of attentional resources using a flanker paradigm. While previous literature has shown that dynamic attentional maps adapt to saccade error signals through saccadic adaptation, our data provides evidence that static attentional maps can be adapted as well. These results suggest that static and dynamic attentional mechanisms may share spatiotopic maps of the visual field that are additionally controlled by the oculomotor system. Further research is necessary to examine the nature of these links and study its underlying neurophysiology.

## Author Contributions

FW programmed the experiments, helped with data collection, and wrote manuscript. All authors contributed to the experimental design, data analysis, and editing the manuscript.

## Conflict of Interest Statement

The authors declare that the research was conducted in the absence of any commercial or financial relationships that could be construed as a potential conflict of interest.
